# Plasma medicine for neuroscience—an introduction

**DOI:** 10.1186/s41016-019-0172-9

**Published:** 2019-10-19

**Authors:** Xu Yan, Jiting Ouyang, Chenyang Zhang, Zhongfang Shi, Boran Wang, Kostya ( Ken) Ostrikov

**Affiliations:** 1 Beijing Neurosurgical Institute, Capital Medical University, Beijing, 100050 China; 2 School of Physics, Beijing Institute of Technology, Beijing, 100081 China; 3 Beijing Tiantan Hospital, Capital Medical University, Beijing, 100050 China; 4 Institute for Health and Biomedical Innovation and School of Chemistry, Physics and Mechanical Engineering, Queensland University of Technology, Brisbane, 4000 Australia; 5 CSIRO-QUT Joint Sustainable Processes and Devices Laboratory, PO Box 218, Bradfield Road, Lindfield, Sydney, New South Wales 2070 Australia

**Keywords:** Cold plasma, Plasma medicine, Neuroscience, ROS/RNS

## Abstract

Plasma is an ionized gas. It is typically formed at high temperature. As a result of both the development of low-temperature plasma sources and a better understanding of complex plasma phenomena over the last decade, “plasma medicine” has become a booming interdisciplinary research topic of growing importance that explores enormous opportunities at the interface of chemistry, plasma physics, and biomedical sciences with engineering. This review presents the latest development in plasma medicine in the area of the central nervous system and aims to introduce cutting-edge plasma medicine to clinical and translational medical researchers and practitioners.

## Main text

### What is plasma?

If we mention the word “plasma,” the first thing a doctor might think of would be the yellowish liquid component of blood that normally suspends the blood cells in suspension. Of course, that definition is correct. However, a more common description for plasma should be a quasi-neutral gas of neutral and charged particles that exhibit collective behavioral pattern, which is also the most bountiful phase of matter in the vast expanse of the universe.

**Fig. 1:**
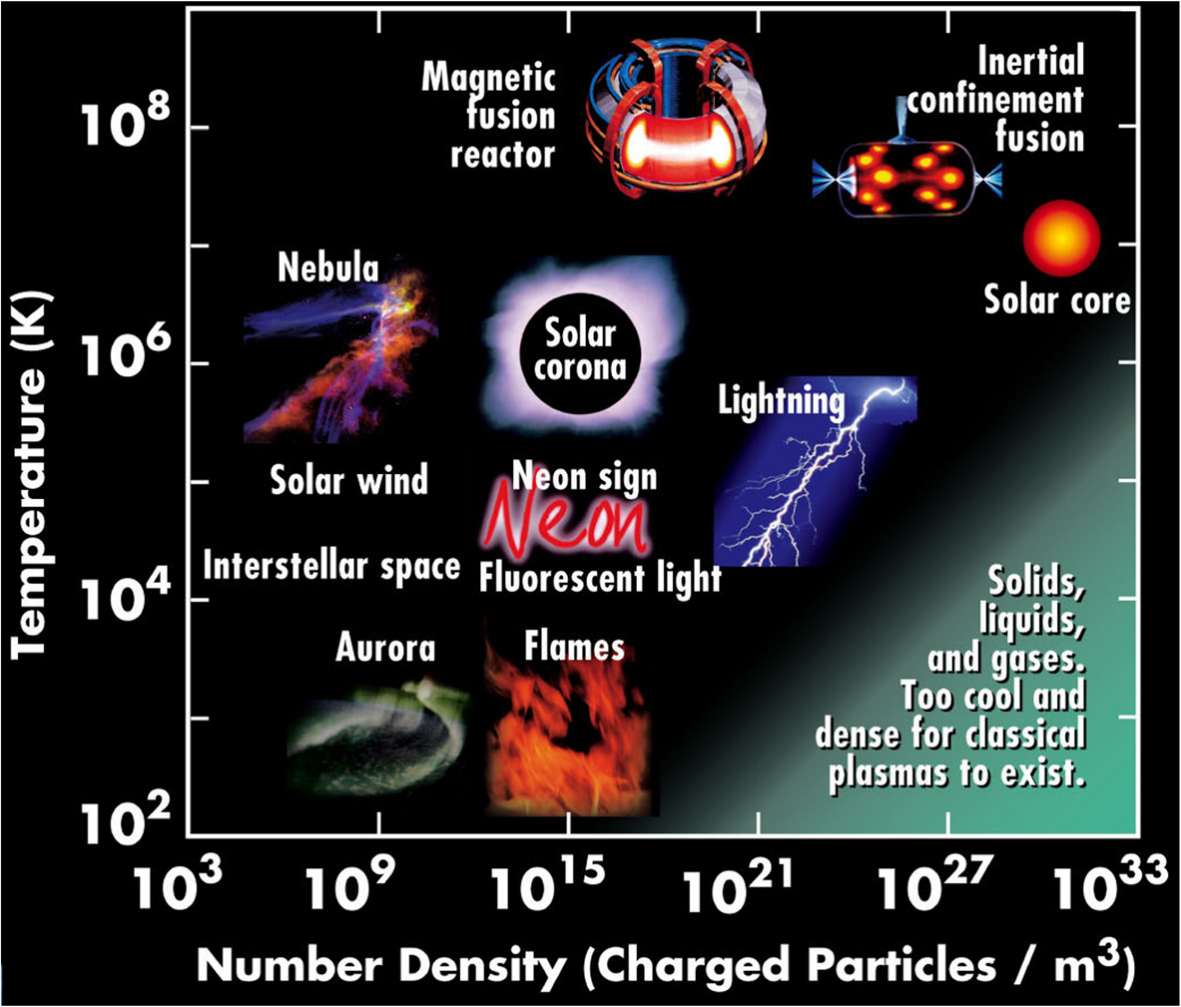
Four states of matter and various plasmas. Plasmas comprise charged and freely moving particles, such as electrons and ions. Plasmas are generated under high temperatures when electrons are detached from neutral atoms. Plasmas are prevalent in nature. For example, stars are mainly plasma. Due to unique physical properties, plasmas are indeed the “fourth state of matter” as distinguished from gases, solids, and liquids. Temperatures and densities of plasma vary extensively (use for reference from “Fusion - Physics of a fundamental energy source, Plasmas - the fourth state of matter, Characteristics of typical plasmas” at http://FusEdWeb.llnl.gov/CPEP/)

From the perspective of physics, plasma is regarded as the fourth state of matter along with solids, gases, and liquids. Plasma is the least recognized, but the most widespread form of the fourth state of matter, comprising more than 99% of the universe. Plasmas are in fact a “cosmic soup” of freely moving and fixed atoms, molecules, ions, and electrons of a variety of densities as well as temperatures ranging from cold to extremely hot (Fig. 1). Plasmas are highly conductive and can respond to magnetic and electric fields; thus, it can serve as an efficient energy source.

**Fig. 2:**
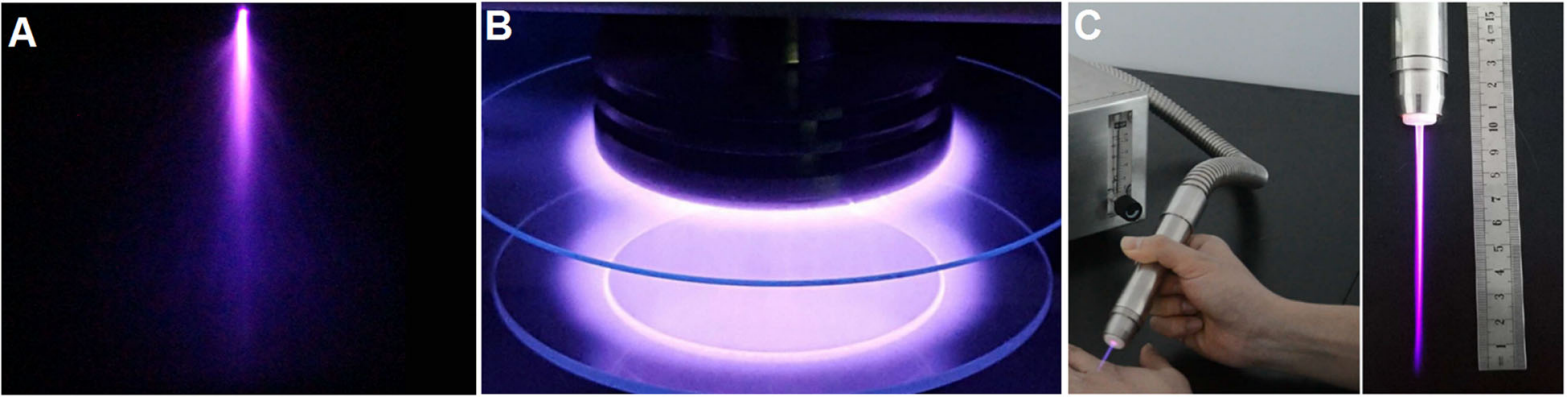
Typical measures to produce cold plasma at atmospheric pressure include corona (**a**), DBD (**b**), and APPJ (**c**)

The majority of plasma forms, such as lightning, stars, and the sun, cannot be easily tested because they are tremendously hot or “superheated” and are difficult to handle. However, recently, researchers have been developing techniques to manufacture non-thermal plasmas that can be operated at atmospheric pressure and room temperature. One of the most important features is the non-equilibrium in that electrons are as hot as 10^4^ K or above, but ions and neutrals are cool at approximately 300 K. The less dense forms of plasma at lower temperature are interrelated to auroras and stellar and solar winds. Moreover, the plasma produced by glow discharge results in the phenomena of colored light in the sky which are Aurora Australis in the southern hemisphere and Aurora Borealis in the northern hemisphere. In the laboratory, we also usually get plasma in the form of gas discharge. Typical electrical discharges that produce cold plasma at atmospheric pressure include corona, dielectric barrier discharge (DBD) and atmospheric-pressure plasma jet (APPJ) [1], and some other plasma sources (Fig. 2).

### Historical perspective of plasma medicine

Some previous applications of plasmas in the medicine industry were mainly on the basis of its thermal effects [2]. It has been decades since the clinical use of thermal plasma, in which plasma is applied in for cauterization as well as blood coagulation, as exemplified in the argon plasma coagulator [3]. In the early and mid-1990s, with the development in the generation of plasma with low temperature, large volume, and atmospheric pressure, most plasma applications in the medicine industry were related to the more gentle and non-thermal effects for simple and convenient use [4].

In 1996, the sterilization effects of cold plasma were demonstrated [5]. Since then, the medical and biological applications of cold plasma have attracted increasing attention. Based on the potential use of plasmas in soldiers’ wounds treatment and sterilization of abiotic and biotic surfaces, in 1997, a proof of principle research program was financially supported by the Physics and Electronics Directorate of the US Air Force Office of Scientific Research (AFOSR), which lasted for over 10 years [4]. In the meantime, comparable studies carried out in Russia indicated that nitric oxide (NO) generated by plasma is of paramount importance in enhancing phagocytosis and speeding up the proliferation of fibroblasts [6]. This was referred to as “plasmadynamic therapy” of wounds by Russian researchers and was demonstrated in experiments both in vivo and in vitro [6]. In 2002 and 2004, researchers from the Netherlands identified the non-aggressiveness of the plasma, which could be applied to detach mammalian cells without leading to necrosis, and they found that in some conditions, low-temperature plasma can result in apoptosis (programmed cell death) [7, 8].

**Fig. 3:**
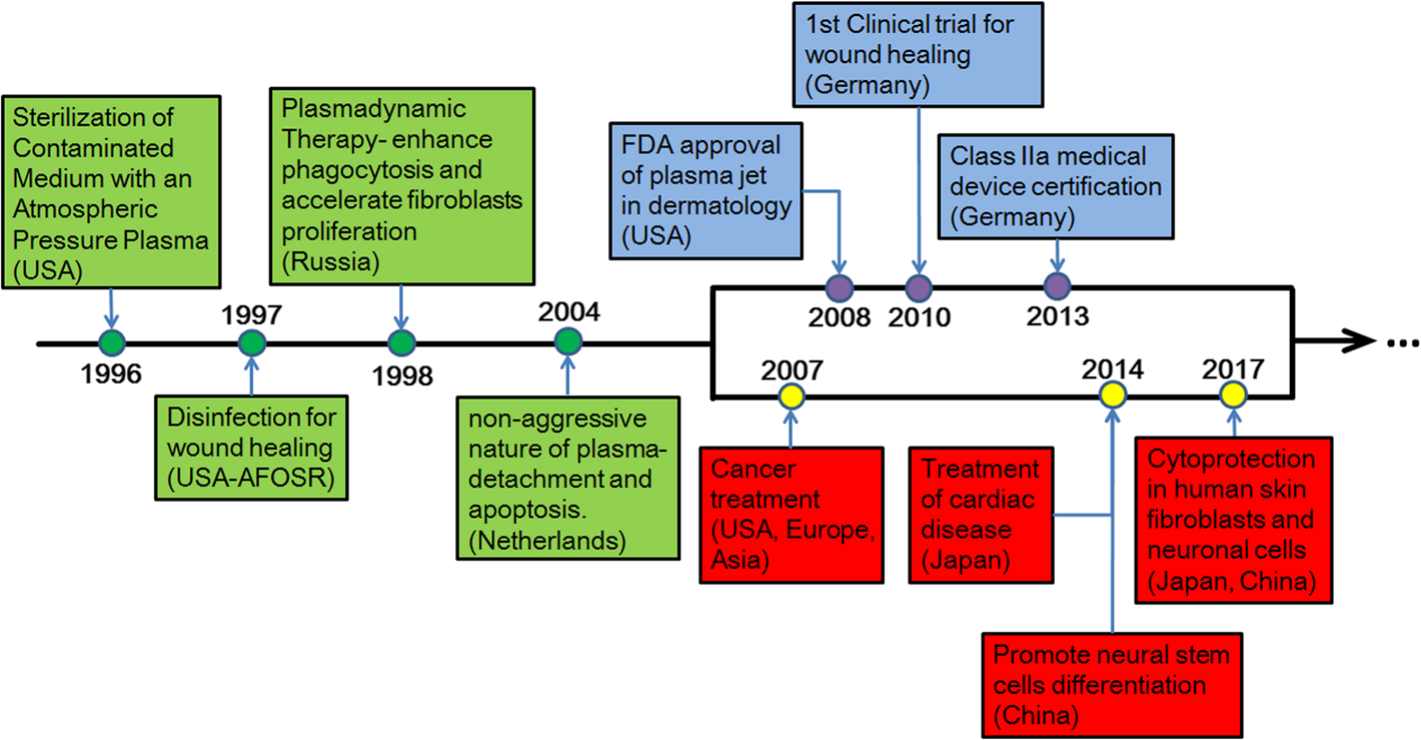
Timeline indicating some of the major breakthroughs in the nascent field of the biomedical applications of plasma at atmospheric pressure and low temperature. This timeline does not indicate the use of thermal plasmas, which have been employed for decades in medical applications that require heat, i.e., blood coagulation and cauterization (after Laroussi [9])

The aforementioned early breakthroughs in plasma studies by researchers paved the way for a nascent multidisciplinary field of study: the biomedical use of plasma at low temperature. By 2007, the International Conference on Plasma Medicine (ICPM), which was the first conference devoted to plasma medicine, was established and held every 2 years. In addition, the first workshop entirely committed to the applications of low-temperature plasma (LTP) in tumorous diseases, the International Workshop on Plasma for Cancer Treatment (IWPCT), was founded in 2014 [4]. Some important milestones were shown as a time line in Fig. 3, indicating the biomedical applications development of LTP, and a nascent field of study known today as “plasma medicine” was gradually formed, which mainly focused on the interaction between LTP and biological tissues, cells, and systems.

### Basic knowledge on biological plasma effects

**Fig. 4:**
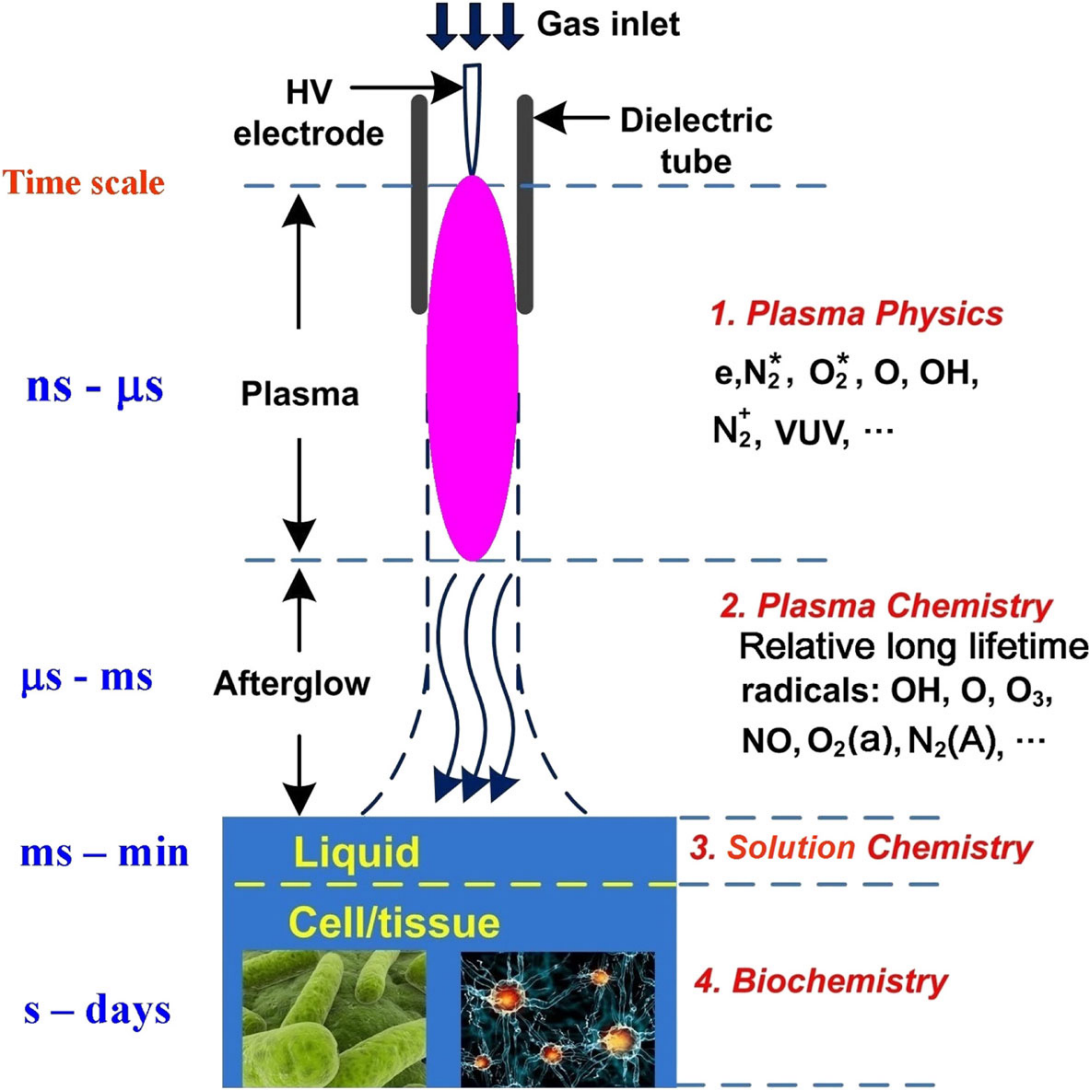
Following Lu et al. [10], the generation, interactions, and transport of reactive species among a variety of states of matter and spatial scales associated with interactions of plasma with biological objects

Plasma that lends itself to biomedical applications is mostly created in the open air. A number of reactive oxygen species (ROS) and reactive nitrogen species (RNS), like O, OH, O_2_^−^, O_2_ (^1^Δ), NO, and H_2_O_2_ (Fig. 4), are produced by the reaction of a small quantity of H_2_O, O_2_, and N_2_ with excited-state species, ions, and electrons in the plasma in the air. RNS and ROS are of importance in oxidation and reduction biochemistry in many living organisms with significant biological implications [11]. A previous publication by Yan et al. in 2012 reported that plasma treatment could lead to ROS and NO accumulation both intracellularly and extracellularly, which demonstrated a direct relationship between plasma physics and biology, as well as medicine [12]. Based on the biomedical interactions of ROS and RNS from plasma, plasma medicine covers a number of applications of LTP in medicine and biology, including sterilization, plasma-aided wound healing, plasma dentistry, plasma pharmacology, plasma oncology, and plasma treatment for implants to enhance biocompatibility. In the next section, the applications of plasma in neuro-protection, differentiation of neural stem cells, and tumors, particularly the glioma, will be summarized.

### Anti-cancer activities of cold plasma-based on the nitrative and/or oxidative stress of plasma

On the bases of the oxidative stress and nitrative stress of plasma treatment on cells or tissues, the latest application that comes under the spotlight is the anti-cancer activities of cold plasma. The first report about the anti-cancer applications and the apoptosis of cancer cells in response to plasma treatment was published by Fridman et al. in 2007 [13]. Since then, a series of studies have confirmed the remarkable anticancer capacity of cold plasma and a number of cancer cell lines have been found vulnerable to CAP treatment, such as skin cancer cell lines [14], hepatoma [15], colorectal cancer cell lines [16], gastric cancer cell lines [17], breast cancer cell lines [18], pancreatic cancer cell lines [19], lung cancer cell lines [20], cervical cancer cell lines [21], head and neck cancer cell lines [22], leukemia [23], and even brain tumors. Glioblastoma is the most prevalent and one of the most aggressive main brain tumors with a devastatingly poor prognosis.

### Plasma treatment of glioblastoma

Cold plasma has been highlighted in the in vitro anti-glioblastoma activities in different kinds of glioblastoma cell lines for many years [24, 25]. Mechanistic research on the anti-glioblastoma activities of plasma has mainly focused on the apoptotic and survival signal pathways mediated by the reactive species from the plasma emissions [26] or the plasma-mediated medium (PAM) [27, 28]. For example, Tanaka et al. found that PAM could downregulate cell survival and proliferation pathways, such as extracellular regulated kinase (ERK), serine-threonine kinase AKT, and mammalian target of rapamycin (mTOR) signaling pathways [29]. Another interesting publication by Kaushik et al. found that plasma-induced reactive species induced cytotoxic RAW 264.7 macrophages to release TNF-α, which could block the growth of cancer cells when cocultured with the T98G human glioblastoma multiforme cell line [29]. Recently, Yan et al. [29] suggested that aquaporin 8 (AQP 8), a kind of H2O2 membrane channel in many types of cells, was closely related to the anticancer capacity of plasma-stimulated medium (PSM) in U87MG glioblastoma cell lines. Inhibiting the expression of AQP8 in U87MG cells played an important role in weakening the anti-glioblastoma effect of PSM.

Some researchers are more focused on the targeted killing effect on glioblastoma cell lines of plasma. Cheng et al. compared the parameters of plasma and the threshold of plasma treatment on a U87 glioblastoma cell line and human astrocytes in normal conditions (E6/E7); these authors found that a plasma treatment that lasted 30 s caused a threefold increase in the death of U87 cells as compared to the E6/E7 cells [26]. Another publication by Tanaka et al. found that, compared with the commercially available normal human astrocytes, U251SP glioblastoma cells were more easily induced to apoptosis by the plasma mediated medium (PAM), which was regulated by downregulating total AKT kinase expression and phosphorylated AKT (Ser473) [30].

The first-line glioblastoma chemotherapeutic drug is temozolomide (TMZ), which is an alkylating agent. Currently, standard GBM care consists of maximal surgical resection, and then radiotherapy along with adjuvant chemotherapy by virtue of TMZ [31]. Recurring issues with present chemotherapeutics employed in GBM treatment are the negative side effects as a result of the high toxic effect of the drugs, and the resistance to the therapy developed over time. In response, combinational therapies are adopted in order to reduce the possibility of drug resistance and the negative side effects related to therapies with a single agent. Recent research from Conway et al. showed that plasma could induce a JNK-, ROS-, and caspase-independent mechanisms of death of the U373MG GBM cell lines, which can be significantly enhanced by virtue of combining low doses of TMZ [32]. Koritzer et al. investigated the effect of plasma on TMZ-sensitive and TMZ-resistant cell lines in glioblastoma cell lines LN18, U87MG, and LN229. The combined treatment of TMZ and plasma resulted in cell cycle arrest and the inhibition of cell growth [33].

**Fig. 5:**
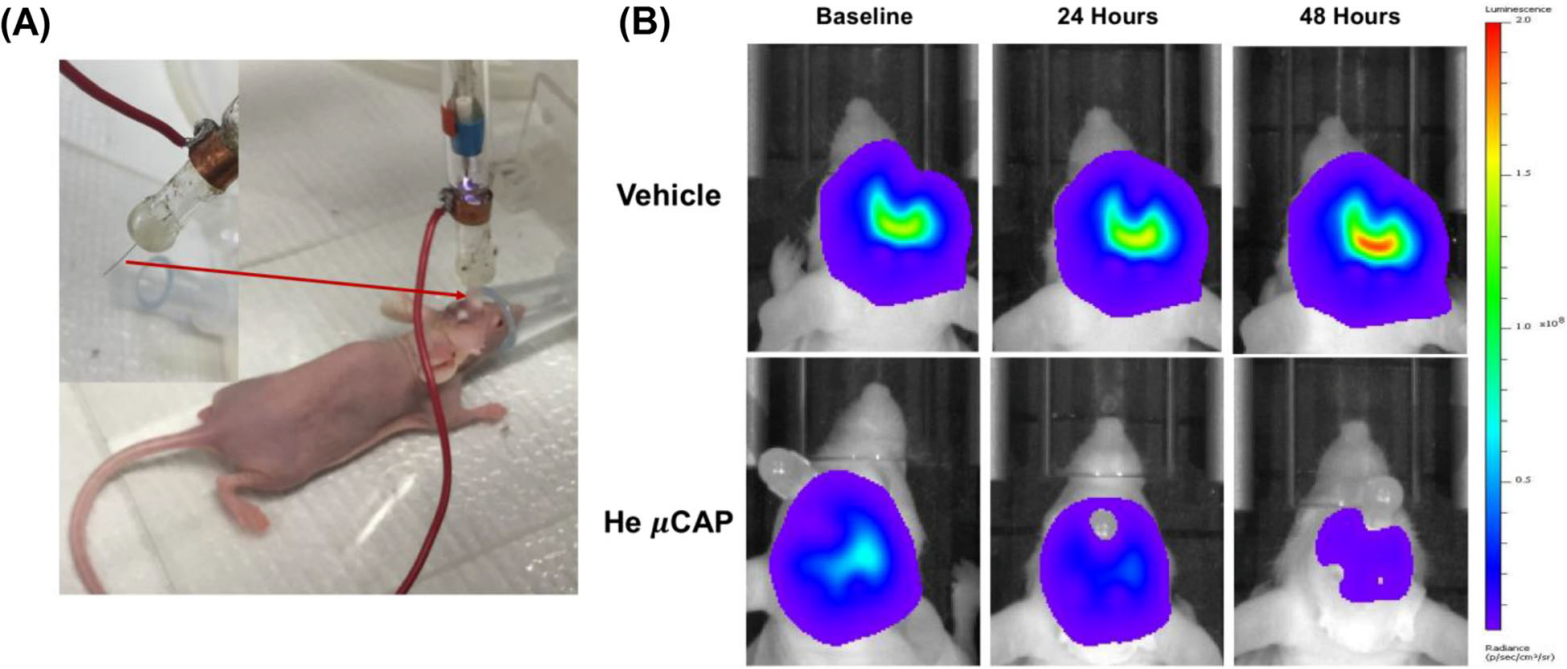
In vivo targeting of glioblastoma tumors by He μCAP [34]. **a** A new μCAP device used in plasma delivery by means of an intracranial endoscopic tube to direct at glioblastoma tumors in the brain of the mouse. **b** Representative bioluminescence images in vivo demonstrating the volume of glioblastoma tumor (such as radiance or light emission) at baseline and 2 days after the treatment of He μCAP or vehicle (helium). Areas of low photon emission are indicated in blue and high are indicated in red

Some researchers have tried to translate the in vitro research about the anti-glioblastoma effect of plasma into animal studies. Research from Yan et al. also demonstrated the anti-glioblastoma effect of plasma both in the U87MG glioblastoma cell line and U87MG xenografts in rat brains. This research was based on a novel microsized CAP device (μCAP), of which the inner diameter of quartz tube was only 70 ± 3 μm, so the tube could be implanted into the mouse brain for the in vivo treatment of glioblastoma (Fig. 5). The results showed significant inhibition of tumor volume compared with the control animals [34]. Other research by Vandamme et al. also showed that plasma treatment could reduce subcutaneous glioma xenograft volume and prolong overall survival rate in rats [35].

### Neural stem cells differentiation and neuro-protection of plasma-based on the physiology functions of ROS and RNS from “suitable” low-dose plasma treatment

The RNS and ROS generated by the APPJ treatment have been regarded to associate the biomedical applications with plasma for a long time [36, 37]. Previous researches highlighted the inactivation effects of microorganisms (fungi, viruses, bacteria, etc.), cancer tissues, or cells as a result of the accumulation of nitrative stress and/or oxidative stress mediated by APPJ [10, 11, 38]. Nevertheless, RNS and ROS play more significant physiological roles in the human body [39, 40]. RNS and ROS at physiological concentrations can result in positive physiological advantages, including maintaining homeostasis [41], initiating immune responses [42], and regulating cell differentiation and proliferation [43], by means of functioning as intracellular signaling molecules which are engaged in the pathophysiological and physiological processes.

Among these active species produced by plasma, NO plays a critical role in the central nervous system (CNS). NO is a crucial signaling molecule that regulates a variety of biological processes and plays a double role in the human body, particularly in terms of CNS (i.e., neurotoxicity and neuroprotection). Excessive generation of NO not only induces apoptosis but also is involved in the pathophysiology of neurodegenerative disorders [44]. On the other hand, NO at physiologically low levels can function as a neuroprotective agent in the face of a variety of death challenges by regulation of the downstream signal pathways, like PI3 kinase/Akt and NO-cGMP-PKG pathways [45, 46]. A quantity of pharmacological researches have indicated that gaseous NO and various NO donor drugs have been widely used for a variety of vascular and CNS disorders or as a neuroprotective agent during brain injuries [46–51]. Furthermore, in vivo researches suggested that NO could also function as a powerful cerebral vasodilator to improve the supply of cerebral blood flow (CBF) into the infarcted area during hypoxic or ischemic brain injuries [52, 53].

**Fig. 6:**
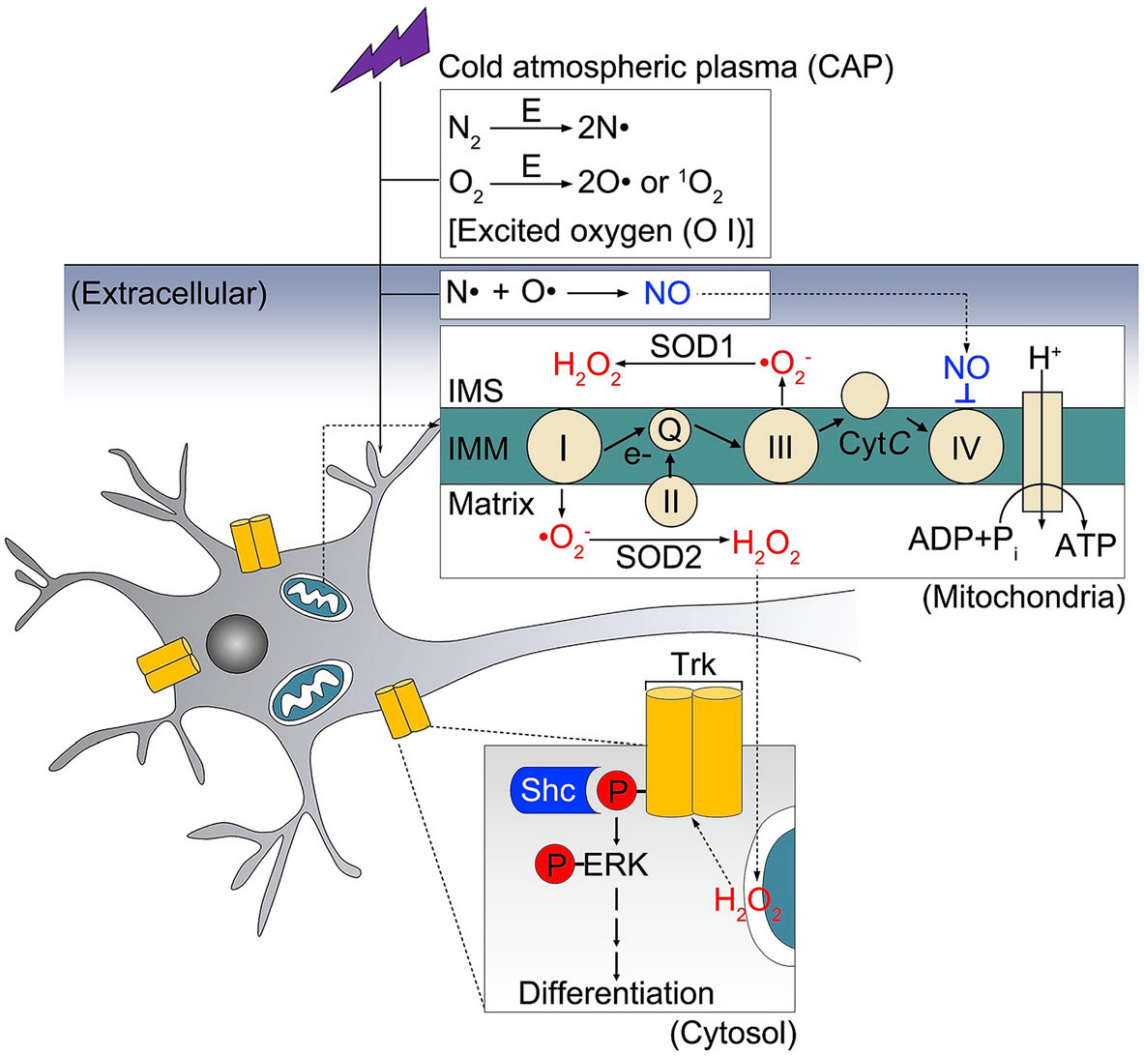
Following Jang et al. regarding the neural differentiation mechanism of cold plasma. Plasma treatment directly induced an increase in extracellular NO and targeted mitochondria. NO contends with O_2_ for binding to the active site of the cytochrome c oxidase, mitochondrial electron transport chain complex IV, and reversibly impeding its activity. Mitochondrial •O_2_^−^ is generated by virtue of an electron reduction of O_2_ by means of obstructing electron transfer in the electron transport chain. Then, mitochondrial •O_2_^−^ further leads to the production of intracellular H_2_O_2_, which could activate the Trk/Ras/ERK signaling pathway in specific by playing the role of an intracellular messenger, and finally resulted in neural differentiation

Therefore, some researchers focus on the potential use of plasma on CNS disorders. Xiong et al. first reported that cold plasma could selectively promote the differentiation of neural stem cells into neuronal cells, which was regulated by the nitric oxide (NO) generation of plasma emissions [54]. The mechanism of the neuronal differentiation effect of plasma on neural stem cells was detailed by Jang et al. These authors found that plasma treatment could increase the extracellular NO concentration, which induced reversible inhibition of mitochondrial complex IV and produced large amounts of superoxide radical (•O_2_^−^). Cytosolic hydrogen peroxide, which is generated by •O_2_^−^ dismutation, activated the Trk/Ras/ERK signaling pathway in specific by playing the role of an intracellular messenger. In addition, the authors also proved the differentiation effect of plasma in the living zebrafish in vivo (Fig. 6) [55].

Then, some researchers focused on the treatment effect of plasma using in vitro disease models of the central nervous system (CNS). Oxidative stress-induced cell damage has long been implicated in both the physiological process of aging and neurodegenerative disorders, including Alzheimer’s disease (AD) and Parkinson’s disease (PD). Yan et al. pretreated SH-SY5Y cells, which is a cell line commonly used for neurotoxicity or neuroprotection research in vitro by an atmospheric pressure plasma jet (APPJ), and then subjected the cells to oxidative stress injuries. These authors found that pretreatment by APPJ could rescue SH-SY5Y cells from the harm of oxidative stress by decreasing cell apoptosis, which could be associated with the reactive nitrogen species that were induced by the treatment of APPJ [56]. Two researchers from the same group also found that APPJ pretreatment could protect against glucose deprivation (to stimulate glucose metabolism dysfunction diseases in CNS) [57] and hypoxia-induced injury (to simulate ischemia diseases in CNS, especially stroke) [58] in the SH-SY5Y cells. In addition, the authors also found that APPJ treatment could reduce the hypoxia microenvironment induced by an over-proliferation of cells, using a real-time cell viability recording system, namely, the electric cell-substrate impedance sensing system [58], suggesting a new mechanism of the anti-tumor capacity of APPJ that deserves further investigation.

## Conclusions

Plasma medicine is a multidisciplinary field of study, coupling plasma physics, medicine, biology, plasma chemistry, and engineering, grown from studies in the application of atmospheric plasmas under low-temperature (or cold) in biomedicine. The current review summarized some applications of plasma medicine in neuroscience, especially in anti-glioblastoma, neuro-differentiation, and neuroprotection, based on the biological production of RNS and ROS in response to plasma emissions. In future studies, identifying key plasma-generated ROS/RNS and tracking their influence on different cells in the brain and even on neoplastic cells will be of growing importance. Issues like the origin of these species, their transportation to and within cells, what reactions are involved in these processes, and ultimately, how their effects permeate other cells will be of vital importance to be studied in the future. The current level of studies suffices to prove that plasmas can (eventually) achieve special use in the CNS in direct molecular transportation of medical substances. By controlling the parameters, plasmas may ultimately be able to produce specific species that propagate into the cellular tissues and cause the desired biological and medical effects.

## Data Availability

The datasets used and/or analyzed during the current study are available from the corresponding author on reasonable request.

## Abbreviations

AD: Alzheimer’s disease
AFOSR: Air Force Office of Scientific Research
APPJ: Atmospheric-pressure plasma jet
AQP 8: Aquaporin 8
CBF: Cerebral blood flow
CNS: Central nervous system
DBD: Dielectric barrier discharge
ECIS: Electric cell-substrate impedance sensing
ERK: Extracellular regulated kinase
ICPM: International Conference on Plasma Medicine
IWPCT: International Workshop on Plasma for Cancer Treatment
LTP: Low-temperature plasma
mTOR: Mammalian target of rapamycin
NO: Nitric oxide
PAM: Plasma-mediated medium
PD: Parkinson’s disease
PSM: Plasma-stimulated medium
RNS: Reactive nitrogen species
ROS: Reactive oxygen species
TMZ: Temozolomide
μCAP: Microsized CAP device
